# American Medical Association

**Published:** 1855-05

**Authors:** 


**American Medical Association.**

This Association commenced its eighth Annual Session on the first day of this month at Philadelphia, in the spacious Musical Fund Hall of that City. We have received a sketch of the first day's proceedings, for which we are indebted to the Delegate from the College, Prof J. E. Sanborn.

The Association was called to order by the President, Chas. A. Pope, M. D., of St. Louis, Mo.; Dr. West, of Philadelphia, and Dr. LeMoine, of St Louis, Secretaries.

Dr. Hays, on behalf of the profession of Philadelphia, welcomed the members of the Association to that city in a very cordial manner.

The roll was then called, and, from the length of the list, the Convention was a very full one.

The President, by invitation, addressed the Association. We refrain from any remarks upon the merits of this address, as we shall publish it entire in a future number.

Dr. Hays invited the members to the Pennsylvania Hospital, omnibuses being then in readiness.

A nominating Committee, consisting of one from each State, was then chosen.

VISITATIONS.

They visited Independence Hall, where they were received by Hon. R. T. Conrad, at 4, p. m., of the Fairmount and Girard College. Invitations were given to visit other public Institutions.

A resolution, to go into the election of officers, was laid upon the table.

Invitations were tendered to meet next year at Chicago, Detroit and Nashville.

The Prize Essay was awarded to Dr. J. Trask, N. Y. Subject, "Statistics Placenta Previa."

Committee on Epidemics of Missouri, Iowa and Wisconsin, reported. Ordered to be printed.

A report on the "Hydrometrical state of the atmosphere in various localities and its influence on health," was made by Dr. Hunt.

Adjourned to meet to-morrow.

The evening was spent at the houses of Drs. Hodge, Norris and Bache, where repasts were prepared.

